# Obesity in Repaired Tetralogy of Fallot: Implications for Symptoms, Right Ventricular Assessment, and Pulmonary Valve Replacement

**DOI:** 10.3390/jcm15218487

**Published:** 2026-11-01

**Authors:** Giovanni Di Salvo, Elettra Pomiato, Francesca Marino, Yara Alras, Ayat Shoaib Shah, Francesco Prati, Domenico Galzerano, Valeria Pergola

**Affiliations:** 1 Pediatric Cardiology and Adult Congenital Heart Disease, Department of Child and Woman Health, University of Padua, 35128 Padua, Italy; 2 Department of Clinical Medicine and Surgery, Section of Endocrinology, University ‘Federico II’ of Naples, 80131 Naples, Italy; 3 College of Medicine, Al Faisal Medical University, Riyadh 11533, Saudi Arabia; yalras@alfaisal.edu (Y.A.); asshah@alfaisal.edu (A.S.S.); 4 Heart Center, King Faisal Specialist Hospital and Research Center, Riyadh 11211, Saudi Arabia

**Keywords:** tetralogy of Fallot, obesity, right ventricle, pulmonary regurgitation, cardiac magnetic resonance, body surface area, exercise capacity, pulmonary valve replacement

## Abstract

Obesity complicates assessment after repair of tetralogy of Fallot (rTOF) because it can influence symptoms, exercise performance, ventricular remodeling, and the normalization of ventricular measurements. This narrative review examines the direct rTOF evidence, almost all observational, and its implications for pulmonary valve replacement (PVR) decision-making. Obesity is present in approximately 15–20% of patients (12–26% across the cohorts reviewed). In studies using absolute, height-indexed, or ideal-BSA-indexed volumes, obesity was associated with larger biventricular volumes and lower ventricular ejection fractions, and the RV volume association persisted after adjustment for PR in one study. These findings have implications for the interpretation of cardiac findings in patients with moderate-to-severe PR after rTOF. Decisions are best made by integrating all findings, with extra attention to patients with obesity. We propose an integrated assessment of symptoms, rhythms, exercise findings, and ventricular measurements.

## 1. Introduction

Long-term assessment after repair of tetralogy of Fallot (rTOF) is focused on residual pulmonary valve and right ventricular outflow tract (RVOT) disease, ventricular remodeling and dysfunction, and arrhythmias [1,2,3,4]. These abnormalities, together with symptoms and exercise performance, guide risk assessment and pulmonary valve replacement (PVR) decisions [1,2]. Obesity introduces an additional concern because it may affect the clinical findings and, through indexing to body surface area (BSA), the interpretation of true ventricular volumes.

In a population-based Danish cohort of adults with congenital heart disease, obesity was as prevalent as in the matched controls (16% versus 15%), although the patients had become obese at a younger age [5]. The direct rTOF cohorts demonstrated that excess weight is clinically relevant, although its prevalence depends on age, selection, and definition. Among 260 pediatric patients referred for cardiac magnetic resonance (CMR), Fogel et al. identified obesity in 32 (12.3%) and overweight in 48 (18.5%) [6]. In Egbe et al.’s cohort of 461 adults with moderate/severe pulmonary regurgitation (PR), 119 (26%) had obesity [7]. The higher prevalence in the adult cohort is consistent with an increase with age, although the cohorts differed in their selection (referral for CMR versus moderate/severe PR).

Prognostic evidence of obesity on mortality in rTOF also comes directly from rTOF cohorts. Mayourian et al. [8] studied 1552 patients and identified obesity as an independent predictor of all-cause mortality in the model development cohort (adjusted hazard ratio, 1.70; 95% confidence interval, 1.01–2.86; the lower limit is close to unity). Obesity was incorporated into an externally validated mortality risk score [8]. Although this observational association does not establish causation or demonstrate that weight reduction improves survival, it supports the relevance of obesity to risk assessment beyond extrapolation from other populations.

Previous reviews have addressed obesity in congenital heart disease as a whole [9,10] or its prevalence and treatment in adults with congenital heart disease [5,11]. This review is restricted to rTOF. The aim of this review is to address five clinical questions: how much ventricular remodeling can be associated with obesity beyond PR; whether evidence supports an obesity-related increase in pulmonary afterload; how symptoms and exercise limitation should be interpreted when obesity and severe PR coexist, with or without arrhythmia; whether actual body surface area (BSA) indexing obscures clinically important RV enlargement, and whether biventricular volumes should be interpreted differently in obese patients, for example by using ideal BSA indexing; and how these issues may influence PVR timing. The objective is to connect the quantitative rTOF evidence to clinical interpretation, identify the unresolved questions, and propose an interpretive ground for obese patients with rTOF.

## 2. Literature Search Strategy

A narrative literature search was performed in PubMed/MEDLINE and last updated on 12 October 2026. The search concepts included “tetralogy of Fallot,” “obesity,” “overweight,” “body mass index,” “cardiac magnetic resonance,” “ventricular volume,” “indexing,” “height,” “exercise,” “pulmonary regurgitation,” “pulmonary valve replacement,” “arrhythmia,” and “mortality.” The reference lists of retrieved articles, guidelines, and previous reviews were screened for additional studies, and publications on the prevalence and treatment of obesity in adults with congenital heart disease [5,11] were also considered.

Human original studies directly involving rTOF were prioritized. Mixed congenital heart disease (CHD) studies and general population guidance were retained only where their applicability and limitations were explicit. Conference reports that preceded full publications were not counted as independent evidence. No formal systematic review screening, risk-of-bias scoring, or meta-analysis was undertaken. Almost all the rTOF-specific evidence identified was observational. Table 1 summarizes the clinical implications. Table 2 distinguishes the study populations, PR handling, quantitative findings, and limitations. Table 3 summarizes the functional, rhythm, and clinical outcome studies. We designed a clinical framework on the basis of the available evidence. The framework and the proposed reassessment interval are author interpretations, not validated intervention strategies.

The abbreviations used are defined as follows: BSA, body surface area; CMR, cardiac magnetic resonance; ECG, electrocardiography; EDV, end-diastolic volume; EF, ejection fraction; FMI, fat mass index; HIIT, high-intensity interval training; HRQoL, health-related quality of life; PR, pulmonary regurgitation; PVR, pulmonary valve replacement; RVESVi, right ventricular end-systolic volume index; VO_2_, oxygen uptake.

## 3. Definition and Classification of Overweight and Obesity

Adult body mass index (BMI) categories follow World Health Organization thresholds: overweight is 25.0–29.9 kg/m^2^ and obesity ≥30.0 kg/m^2^ [23]. In children and adolescents, BMI is interpreted relative to age and sex. In the Centers for Disease Control and Prevention (CDC) framework, overweight is from the 85th to <95th percentile and obesity ≥95th percentile [24]; the WHO reference for 5–19-year-olds defines overweight as BMI-for-age >+1 standard deviation and obesity as >+2 standard deviations [25]. Because the studies reviewed used different age ranges and classification systems, the cohorts were not reclassified; the definitions used in each key study are summarized in Table 2. However, BMI is a screening measure rather than a direct measure of adiposity and does not distinguish fat from lean mass. Chang et al. included the fat mass index as well as BMI in their rTOF study and found that exercise performance, expressed relative to the predicted values and in absolute terms, differed between adiposity groups [17]. Detailed ectopic fat imaging is outside the scope of this review because no validated rTOF-specific threshold would guide PVR decisions.

## 4. Obesity Across the rTOF Life Course

Prevalence of overweight and obesity in rTOF appears to increase with age, although the cohorts differed in their selection and definition. In pediatric cohorts, obesity was identified in 12.3% of 260 patients referred for CMR [6], and overweight/obesity in 12 of 85 children with TOF variants (14%) [19]; in an adult cohort with moderate/severe PR, obesity was present in 26% [7]. In 137 patients with TOF, Briston et al. observed convergence of childhood BMI with population norms by late adolescence [18]. Clinical correlates of excess weight were reported separately: a higher adult BMI was associated with a worse New York Heart Association (NYHA) class despite a similar left ventricular (LV) ejection fraction (LVEF) [18], and overweight/obesity in children with lower physical and social health-related quality-of-life scores [19]. These observations support assessment of weight throughout congenital follow-up without assuming identical prevalence or effects across ages.

## 5. Cardiac Associations in rTOF: Comparison of Cardiac Findings in Obese and Non-Obese Patients with rTOF

### 5.1. Quantitative Ventricular Remodeling and Myocardial Mechanics

CMR studies suggest that obesity in repaired tetralogy of Fallot (rTOF) may be associated with true adverse biventricular remodeling, not merely with an artifact of body size indexing. Fogel et al. [6] reported a greater ventricular mass indexed to height^2·7^ across weight categories (obese, 40.4 ± 14.4; overweight, 38.3 ± 13.0; normal weight, 35.2 ± 11.6 g/m^2·7^; *p* = 0.046). Their reported actual-BSA-indexed RVEDV differed across groups (106.3 ± 26.4, 123.1 ± 49.4, and 121.3 ± 37.7 mL/m^2^, respectively; *p* = 0.032), but did not establish a greater RV volume in obesity; because indexing was to the actual rather than ideal BSA, the larger body size of the obese group may have lowered the indexed values and led to underestimation of any obesity effect. Maskatia et al. [12] compared 36 obese with 72 normal-weight patients and found a greater RVEDV indexed to height (160 ± 59 versus 135 ± 41 mL/m; *p* = 0.015) and to ideal BSA (166 ± 55 versus 144 ± 38 mL/m^2^; *p* = 0.020), accompanied by a lower RVEF (46 ± 9% versus 51 ± 7%; *p* = 0.003) and LVEF (57 ± 9% versus 61 ± 6%; *p* = 0.017). These between-group differences amount to 25 mL/m, 22 mL/m^2^, and five percentage points, respectively.

Simpson et al. [16] evaluated 86 patients with transannular patch repair. The absolute RVEDV was larger across increasing weight categories (underweight, 180 [122–220]; appropriate weight, 200 [180–236]; overweight, 222 [187–250]; and obese, 258 [212–275] mL; *p* = 0.024). The ideal-BSA-indexed RVEDVi also varied numerically (141, 137, 148, and 160 mL/m^2^, respectively), but did not differ significantly (*p* = 0.207). The ventricular ejection fractions were similar across categories, whereas the LV circumferential strain differed. These findings should not be described as demonstrating an opposite biological effect of obesity: absolute and indexed volumes address different questions, and indexing may modify the apparent direction or magnitude of associations.

Reindexing to the ideal BSA changed the classification relative to the volumetric PVR criteria applied in that study: 11 additional overweight/obese patients (31% of that subgroup) met the criteria, while three underweight patients (38% of that subgroup) no longer did [16]. This is evidence of altered classification, not proof that alternative indexing improves treatment outcomes.

Aly et al. [13] compared 32 obese with 64 age- and sex-matched normal-weight children and adolescents. The actual-BSA-indexed median RVEDVi was 165 versus 157 mL/m^2^ (*p* = 0.20), and the RVESVi was 91 versus 85 mL/m^2^ (*p* = 0.30), for between-group differences of only 8 and 6 mL/m^2^. With ideal BSA indexing, the corresponding values were 199 versus 153 mL/m^2^ for the RVEDVi (*p* < 0.0001) and 113 versus 84 mL/m^2^ for the RVESVi (*p* = 0.0001): differences of 46 and 29 mL/m^2^. The RVEDV and RVESV indexed to height were also greater (184 versus 151 mL/m and 105 versus 80 mL/m, respectively). Obesity was associated with a lower RVEF (45% versus 52%), lower LVEF (52% versus 56%), and greater ideal-BSA-indexed LV mass (76 versus 50 g/m^2^; all *p* < 0.0001). The RV volume associations persisted after adjustment for the PR fraction or regurgitant volume; notably, the PR fraction was 34% in the obese group versus 41% in the controls (*p* = 0.03), so the PR severity should not be described as identical. These results support a clinically relevant obesity-associated biventricular phenotype, while not proving a causal, individually quantifiable obesity contribution.

### 5.2. How Much Enlargement Can Be Attributed to Obesity?

Severe PR is a major cause of RV volume loading in rTOF [1], but it is not necessarily the only determinant of the observed ventricular phenotype. Across Maskatia and Aly’s studies, obesity was associated with larger RV volumes under appropriate alternative normalization and with lower biventricular ejection fractions [12,13]. The associations between obesity and increased RV volumes in Aly persisted after adjustment for the PR fraction or regurgitant volume [13], suggesting that the measured PR severity alone does not fully account for the findings. These observations should not be minimized as a mere indexing nuisance. At the same time, they do not establish how much of an individual patient’s RV enlargement is caused by obesity rather than PR, residual obstruction, ventricular interaction, or other factors. Neither the study-level difference in RVEDVi nor that in RVESVi is a validated volume to subtract from an individual measurement.

The clinical task is to assess the complete ventricular phenotype—including RVESVi, RVEDVi, RVEF, LVEDVi, LV mass, LVEF, PR severity, and longitudinal change—rather than to calculate a hypothetical obesity-corrected RV volume. Obesity may be a modifiable contributor to cardiovascular risk, but evidence that intentional weight reduction reverses RV enlargement or improves PVR-related outcomes in rTOF is lacking. No rTOF-specific evidence establishes obesity as a cause of increased pulmonary vascular afterload. Residual RVOT or branch pulmonary artery obstruction should be assessed on its own merits. Recognition of obesity-associated remodeling should encourage comprehensive care without postponing an otherwise indicated PVR.

### 5.3. Tachyarrhythmias and Mortality: The Role of Obesity

In a multicenter CMR cohort of 319 patients with rTOF, Beurskens et al. identified a higher BMI as an independent marker of sustained ventricular tachyarrhythmia (hazard ratio, 1.8 per 5 kg/m^2^ increase; *p* = 0.02) [3]. A higher BMI also predicted atrial tachyarrhythmia (hazard ratio, 1.76 per 5 kg/m^2^ increase) [3]. These are associations with continuously measured BMI rather than proof of a causal obesity effect.

The mortality model developed by Mayourian et al. included obesity, age at CMR, repair type, RV end-systolic volume index, and biventricular global function index. Model discrimination was maintained during external validation (concordance index, 0.80) [8]. These observations support consideration of obesity alongside established cardiac risk markers. They do not demonstrate that intentional weight loss reduces arrhythmia or mortality.

## 6. Interpreting Symptoms and Exercise Performance

### 6.1. Severe PR Does Not Uniformly Determine Exercise Capacity

Exercise-related changes in loading complicate inference from resting PR severity. Roest et al. [14] observed a reduction in PR volume during submaximal exercise (27 ± 17 to 23 ± 15 mL/m^2^; *p* = 0.012), while the RVEDVi increased from 132 ± 36 to 137 ± 38 mL/m^2^ (*p* = 0.041). An increase in end-diastolic volume during exercise is not intrinsically abnormal. Rather, the abnormal response described relative to the controls concerned the limited RV contractile reserve: the RVESV did not decrease significantly and the RVEF did not increase significantly. This study did not directly test an obesity-specific effect. Reduced PR during exercise does not mean that chronic PR is clinically unimportant.

O’Meagher et al. compared patients with an RVEDV index below versus above 150 mL/m^2^. Exercise performance did not differ significantly: the predicted peak oxygen uptake was 84 ± 17% versus 87 ± 12%, and the predicted peak work was 90 ± 17% versus 89 ± 11% [26]. This illustrates that substantial RV enlargement can coexist with relatively preserved exercise performance; it is not a reason to disregard objective progression or other intervention indications.

Percutaneous pulmonary valve implantation (PPVI) data similarly show that eliminating predominantly regurgitant lesions does not consistently increase peak oxygen uptake (VO_2_). Lurz et al. reported values of 25 ± 8, 25 ± 8, and 26 ± 9 mL/kg/min before, early after, and one year after PPVI in the PR subgroup (*p* = 0.48) [15]. An earlier PPVI study also found exercise improvement predominantly after relief of stenosis [22]. These mixed CHD cohorts should not be described as exclusively rTOF, and absence of peak-VO_2_ improvement does not exclude other benefits of valve intervention.

### 6.2. Obesity as a Contributor to Functional Limitation

Aly et al. [13] reported a lower median peak VO_2_ (31 versus 38 mL/kg/min; *p* < 0.0001) and anaerobic threshold VO_2_ (22 versus 30 mL/kg/min; *p* < 0.0001) in obese versus normal-weight patients with rTOF. The peak workload did not differ significantly. Importantly, the reported adjustment for PR fraction or regurgitant volume concerned the RV volume analyses; it should not be interpreted as proof that every difference in VO_2_ persisted independently after adjustment for PR. Exercise limitation may reflect obesity, PR, ventricular dysfunction, deconditioning, chronotropic abnormalities, and other factors. Weight-normalized VO_2_ is also affected by the body mass denominator.

Quantitative interpretation must account for measurement normalization. In 185 adolescents with rTOF, Chang et al. reported a lower predicted peak VO_2_ in the group with excess adiposity by fat mass index (60.04 ± 14.83% versus 69.08 ± 15.18%; *p* < 0.001), but a higher absolute peak VO_2_ (1590.59 ± 582.92 versus 1198.67 ± 439.11 mL/min; *p* < 0.001) [17]. Thus, reduced relative performance does not necessarily imply lower absolute oxygen consumption. This study did not establish a comparison restricted to matched obese and lean patients with severe PR.

In an obese patient with severe PR, symptoms and CPET should therefore be interpreted along with ventricular function, serial remodeling, rhythm status, and residual lesions. Because a higher BMI was associated with sustained ventricular and atrial tachyarrhythmia [3], symptom evaluation in an obese patient should include a rhythm assessment (electrocardiography and, where appropriate, ambulatory ECG monitoring). Obesity may contribute through body mass and fitness, but the available data do not assign symptom causation in an individual. The absolute VO_2_, weight-normalized VO_2_, predicted values using the stated reference equation, test effort, and longitudinal changes should be reviewed together [2,17].

### 6.3. Exercise Training Evidence

Schuermans et al. synthesized 10 exercise training studies involving 208 patients with rTOF. The pooled randomized data showed an improved peak VO_2_ of 3.1 mL/kg/min (95% confidence interval, 0.76–5.36), without reported serious exercise-related adverse events [20]. The home-based, monitored high-intensity interval training (HIIT) trial by van Genuchten et al. also reported improved fitness [21]. These studies support individualized exercise for functional benefit but are not obesity-specific weight loss trials and do not demonstrate improved PVR-related outcomes or survival after weight reduction.

## 7. Ventricular Indexing and PVR Decision-Making

In asymptomatic patients with moderate-to-severe PR, an RVESVi above 80 mL/m^2^ is one of the parameters considered for PVR [2] (see Section 7.3). Because this threshold was derived with conventional BSA indexing, the choice of indexing matters most for RVESVi.

### 7.1. Lessons from LV Mass Indexing

Ventricular volumes and cardiac output track lean mass and metabolic demand more closely than adipose mass, so indexing to the actual BSA in obesity tends to over-normalize [27]. Because adipose tissue is less metabolically active than lean tissue, cardiac output and ventricular size scale more closely with fat-free mass than with total body mass, whereas the BSA calculated from actual weight includes the excess adipose mass. This is a physiological relationship rather than a validated rTOF-specific conversion, and the share of ventricular volume explained by lean mass has not been quantified in rTOF. Actual BSA indexing incorporates excess weight into the denominator and can obscure obesity-associated LV hypertrophy. The 2018 ESC/ESH hypertension guideline specifies LV mass indexed to height^2·7^ and permits BSA normalization in normal-weight patients [28]. This is a relevant precedent: body size normalization should not automatically remove remodeling associated with obesity. However, mass and volume are different measurements, and LV guidance does not establish a height exponent or intervention threshold for RV volumes.

### 7.2. RV Indexing and Clinical Risk

Simpson et al. [16], in 86 patients with transannular patch rTOF, demonstrated that ideal BSA reindexing can change the classification compared to previously published volumetric PVR criteria: 11 additional overweight/obese patients (31% of that subgroup) crossed the criteria, whereas three underweight patients (38% of that subgroup) no longer did. These are classification changes, not validated indications for intervention or evidence of improved outcomes. RVESVi deserves special attention because it reflects end-systolic remodeling and is included in contemporary PVR decision criteria.

Egbe et al. [7] studied 461 adults with rTOF and moderate/severe PR (119 with obesity). Across the entire cohort, the mean RVEDVi was 143 ± 37 mL/m^2^ with the actual BSA and 168 ± 35 mL/m^2^ with the idealized BSA; the mean RVESVi was 71 ± 22 and 83 ± 28 mL/m^2^, respectively. These are within-cohort indexing comparisons, NOT obese versus non-obese differences. Idealized BSA indexing improved discrimination for NYHA classes III–IV (AUC: 0.652 versus 0.619) and cardiovascular adverse events (c-statistic: 0.667 versus 0.641), with most of the improvement observed among obese patients. The ideal-BSA-indexed RVESVi was retained in the selected adverse-event model (HR, 1.04 per 10 mL/m^2^; *p* = 0.004). The study was retrospective and did not establish that alternative indexing or earlier PVR improves outcomes.

Actual BSA indexing can therefore mask clinically relevant enlargement in obesity. One should report the absolute RV volumes, conventional RVEDVi and RVESVi, and complementary height- or ideal-BSA-indexed measurements when the findings are discordant, together with the ventricular function and serial trends. Alternative denominators are not interchangeable, and thresholds derived with conventional BSA cannot be transferred mechanically to ideal BSA indices [7,12,13,16]. Neither indexing method identifies the cause of ventricular enlargement; no validated arithmetic correction can partition obesity-related from PR-related remodeling.

As an illustration, consider a patient 175 cm tall and weighing 110 kg (BMI, 35.9 kg/m^2^). The BSA by the Mosteller formula is 2.31 m^2^. Using the weight corresponding to a BMI of 25 kg/m^2^ (76.6 kg), the idealized BSA is 1.93 m^2^. An RV end-systolic volume of 165 mL gives 71 mL/m^2^ with the actual BSA and 86 mL/m^2^ with the idealized BSA, on either side of the 80 mL/m^2^ criterion [2]; an RV end-diastolic volume of 260 mL gives 112 and 135 mL/m^2^, respectively. This example is illustrative only and does not define an obesity-specific threshold.

### 7.3. Obesity and the Timing of PVR

The 2025 ACC/AHA Multisociety ACHD guideline [2] recommends PVR for symptomatic adults with moderate-or-greater pulmonary valve dysfunction. For asymptomatic adults with at least moderate dysfunction, PVR is reasonable when at least two specified findings are present: RVESVi > 80 mL/m^2^, RVEDV at least twice LVEDV, RVEF ≤ 46%, LVEF ≤ 50%, or progressive exercise decline. The RVESVi is therefore especially pertinent to the obesity/indexing discussion. The historical RVEDVi thresholds associated with postoperative reverse remodeling should not be confused with an absolute safe limit or with a validated obesity-specific cutoff. The RVEDV/LVEDV ratio removes a common BSA denominator but is not obesity-independent, because obesity may affect both ventricles.

Obesity can influence several elements of PVR assessment in opposing directions: actual BSA indices may conceal RV enlargement, while reduced weight-normalized VO_2_ or dyspnea may not be attributable to PR alone. The 2025 guideline does not weigh the contribution of obesity to RV volume, so interpretation of volumes may become more difficult in obesity, and a clinician must judge whether PR alone accounts for the findings. The clinician must integrate the RVESVi, RVEDVi, biventricular function, PR severity, RVOT anatomy, rhythm, symptoms, exercise capacity, and serial progression. No evidence supports subtracting a presumed obesity-related RV volume, or routinely deferring PVR for weight loss when established indications are present. For stable patients with genuinely uncertain indications, weight management and interval reassessment may be reasonable, but this remains an author-proposed approach rather than a guideline-validated intervention strategy [1,2]. Evidence on reverse remodeling after PVR offers some reassurance for a short evaluation interval. Preoperative thresholds associated with preserved remodeling have been reported: an RVEDVi up to about 160 mL/m^2^ or, better, an RVESVi below 80 mL/m^2^, with remodeling potential possibly lost at an RVESVi of about 95 mL/m^2^ [29,30]; an RVESVi below 120 mL/m^2^ has also been proposed as a favorable cut-off [31]. In an asymptomatic patient with dubious findings about the indication for PVR, a 6-month progression evaluation would therefore not be unreasonable, provided that the RVESVi remains well below these limits.

### 7.4. Weight Management and Reassessment in Uncertain Cases

For clinically stable obese patients with uncertain indications, we propose structured weight management and appropriately prescribed physical activity, followed by reassessment at approximately six months. Exercise training studies support improved fitness [20,21], but do not validate this pre-PVR interval. The strategy applies when symptom attribution is uncertain and there is no established indication requiring intervention; deterioration should prompt earlier review.

The treatment options for obesity in adults with congenital heart disease have been reviewed elsewhere [11]. In rTOF, structured lifestyle intervention and prescribed exercise improve fitness [20,21], but rTOF-specific data on weight loss or timing of PVR are not available. The data on ventricular remodeling come from a randomized controlled trial of 12 weeks of exercise training in children and young adults with repaired TOF or Fontan circulation, in which the rTOF data can be examined separately [32]; it was not a weight loss trial. Whatever strategy is chosen, it should not delay an established indication for PVR.

Baseline and repeat assessments should compare symptoms, CPET, rhythm findings, PR severity, and ventricular measurements, with imaging selected according to the clinical question. One should use comparable test protocols and report absolute as well as normalized values. Weight loss reduces BSA, so RVEDV/BSA may increase despite a stable or lower absolute RVEDV. Likewise, VO_2_ expressed per kilogram can rise partly because body weight falls. These are mathematical denominator effects, not evidence of a change in cardiac function by themselves.

Improvement after weight management would support a potentially modifiable contribution to functional limitation, but would not exclude significant PR or override an independent PVR indication. Persistent limitation would require renewed assessment rather than automatically establish a valve indication. The choice between transcatheter and surgical PVR remains a separate anatomical and procedural decision [1,2].

### 7.5. Selective Role of Right Heart Catheterization

**Figure 1:**
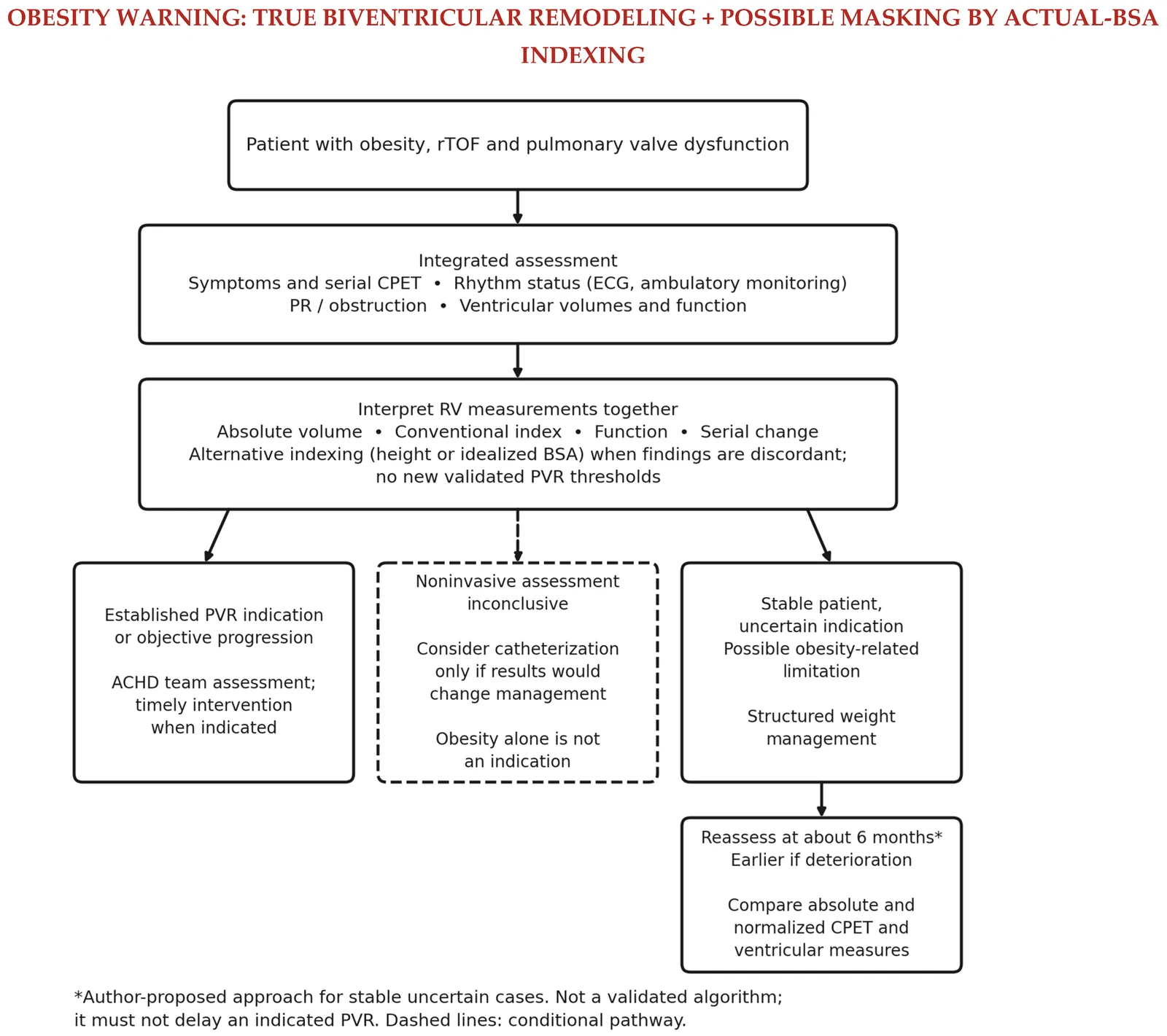
Integrated assessment of obesity in rTOF, including an explicit obesity-related remodeling warning. OBESITY-RELATED REMODELING ALERT: Obesity may contribute to true biventricular enlargement, increased LV mass, and reduced ejection fractions, while actual BSA indexing may conceal clinically relevant RV enlargement. Assess RVESVi, RVEDVi, RVEF, LVEF, LV size/mass, PR, CPET, and longitudinal trends. Do not subtract a hypothetical obesity-related volume. The proposed weight management reassessment applies only to stable uncertain cases and must never delay an indicated PVR. Catheterization remains selective.

Echocardiography and CMR remain the principal investigation tools. Catheterization may be considered when a noninvasive assessment leaves a clinically important hemodynamic question unresolved and invasive measurements are expected to change management [2]. Obesity alone is not a reason for more frequent catheterization (Figure 1).

## 8. Gaps in Knowledge and Future Directions

The obesity–rTOF evidence is almost entirely observational, largely retrospective, and heterogeneous. Only five studies have addressed ventricular volumes in relation to obesity in rTOF [6,7,12,13,16], and longitudinal studies are lacking. Direct studies should compare obese and lean patients with carefully characterized PR, including cohorts without important PR, and report absolute volumes, alternative indices, and longitudinal function. Longitudinal cohorts with repeated imaging would show the natural course and, by modeling PR severity and BMI over time, allow the effects of PR to be separated from those of obesity on ventricular remodeling. Prospective validation should test whether height-based or idealized BSA indexing improves prediction and PVR selection, rather than merely changing the threshold classification [6,7,12,13,16].

Intervention studies should distinguish intentional weight reduction from exercise training and evaluate symptoms, absolute and normalized CPET findings, ventricular remodeling, arrhythmia, and PVR-related outcomes. The proposed six-month reassessment strategy requires evaluation for benefit and safety; no evidence establishes that it improves valve intervention timing. Studies should also determine whether weight reduction modifies the prognostic associations observed in rTOF.

## 9. Conclusions

Obesity in rTOF should be recognized both as a potentially modifiable contributor to adverse biventricular remodeling and as a source of bias in conventional body size indexing. These observational associations cannot quantify the obesity-attributable RV volume in an individual or establish new PVR thresholds. Particular attention should be paid to the RVESVi, RVEDVi, ventricular function, PR severity, symptoms, CPET and longitudinal trends. Weight management may benefit overall health and functional status, but it must not delay an established indication for PVR. Prospective studies are needed to determine whether obesity treatment or alternative indexing improves rTOF-specific outcomes.

## Figures and Tables

**Table 1:** Clinical implications of obesity in rTOF.

Clinical Issue	Evidence	Interpretation
Obesity and ventricular volumes	Obesity was associated with larger biventricular volumes (absolute, height indexed, or ideal BSA indexed) and lower RVEF and LVEF in Maskatia [12] and Aly [13], with RV volume differences persisting after PR adjustment in Aly. Fogel [6], using actual BSA indexing, did not detect a difference.	Obesity is an added risk factor for increased biventricular volumes and decreased RV function, in addition to PR; whether this also applies without PR is not established.
Obesity and prognosis	Obesity entered in an externally validated rTOF mortality model [8].	Relevant risk marker; weight loss benefit for survival remains unproven.
Symptoms and exercise	Obesity-associated RV volume differences persisted after PR adjustment in Aly [13]; CPET differences were not established as PR-adjusted. Exercise and PPVI findings showed variable responses [14,15].	Assess obesity and cardiac abnormalities together; do not infer causation from one measurement.
RV volumes	Actual BSA and alternative indices could classify the same patient differently [7,16].	Review absolute volume, function, alternative indices, and serial change together.
Uncertain PVR indication	Six-month reassessment was a proposal, not trial-supported guidance.	Consider only in stable uncertain cases; do not delay indicated PVR.
Catheterization	Guidance supported selective use after inadequate noninvasive assessment [2].	Obesity alone is not an indication.

**Table 2:** Quantitative evidence on obesity, ventricular remodeling, and RV volume indexing in rTOF.

Study/Cohort	RVEDVi/RVESVi	Biventricular Findings	Interpretation/PR
Fogel 2015 [6]: 260 CMR referrals; 32 obese, 48 overweight	Actual BSA RVEDVi: obese, 106.3 ± 26.4; overweight, 123.1 ± 49.4; normal, 121.3 ± 37.7 mL/m^2^ (*p* = 0.032); no evidence of greater RVEDVi in obese group	Mass/height^2·7^: 40.4 ± 14.4; 38.3 ± 13.0; 35.2 ± 11.6 g/m^2·7^ (*p* = 0.046); EF similar	Pediatric cohort; PR fraction similar; actual BSA comparisons may obscure effects
Maskatia 2013 [12]: 36 obese vs. 72 controls	RVEDV/height: 160 ± 59 vs. 135 ± 41 mL/m; ideal BSA RVEDVi: 166 ± 55 vs. 144 ± 38 mL/m^2^	RVEF: 46 ± 9% vs. 51 ± 7%; LVEF: 57 ± 9% vs. 61 ± 6%	Between-group obesity association; no individual causal partition
Simpson 2018 [16]: 86 transannular patch rTOF	Absolute RVEDV by weight category: 180, 200, 222, 258 mL; ideal BSA RVEDVi: 141, 137, 148, 160 mL/m^2^ (*p* = 0.207)	EF not different; LV circumferential strain differed	11 additional overweight/obese (31%) met study PVR criteria after reindexing; not proof of benefit
Aly 2020 [13]: 32 obese vs. 64 matched controls	Actual BSA RVEDVi: 165 vs. 157; RVESVi: 91 vs. 85 mL/m^2^; ideal BSA RVEDVi: 199 vs. 153; RVESVi: 113 vs. 84 mL/m^2^	RVEF: 45 vs. 52%; LVEF: 52 vs. 56%; LV mass/ideal BSA: 76 vs. 50 g/m^2^	Medians; RV volume associations persisted after PR adjustment; PR fraction 34 vs. 41%
Egbe 2024 [7]: 461 adults; 119 obese	Whole-cohort actual vs. ideal BSA: RVEDVi, 143 ± 37 vs. 168 ± 35; RVESVi, 71 ± 22 vs. 83 ± 28 mL/m^2^	NYHA AUC: 0.619 vs. 0.652; event c-statistic: 0.641 vs. 0.667	Within-cohort indexing comparison, NOT obese vs. non-obese volume difference; moderate/severe PR

**Table 3:** Functional, rhythm, and clinical outcome studies relevant to obesity in rTOF.

Study	Main Findings	Clinical Qualification
Aly 2020 [13]	Peak VO_2_: 31 vs. 38 mL/kg/min; anaerobic threshold VO_2_: 22 vs. 30 mL/kg/min	Exercise outcomes should not be presented as fully PR adjusted; peak workload not significantly different
Chang 2022 [17]	Excess adiposity group: predicted peak VO_2_, 60.04 ± 14.83% vs. 69.08 ± 15.18%; absolute peak VO_2_, 1590.59 ± 582.92 vs. 1198.67 ± 439.11 mL/min	Relative and absolute oxygen uptake may differ in direction
Briston 2017 [18]; Hidestrand 2025 [19]	Higher BMI associated with worse NYHA class; lower physical/social quality-of-life scores in high-BMI pediatric subgroup	Observational and heterogeneous populations
Beurskens 2019 [3]	BMI associated with sustained ventricular tachyarrhythmia (HR 1.8 per 5 kg/m^2^) and atrial tachyarrhythmia (HR 1.76 per 5 kg/m^2^)	Associations do not prove causation
Mayourian 2024 [8]	Obesity associated with mortality (adjusted HR, 1.70; 95% CI, 1.01–2.86); validated risk score	No evidence weight loss improves survival
Schuermans 2023 [20]; van Genuchten 2026 [21]	Exercise training improves fitness; meta-analysis peak VO_2_ + 3.1 mL/kg/min	Not obesity-specific weight loss trials
Lurz 2010/2011 [15,22]	Peak VO_2_ not consistently improved after PPVI for predominantly regurgitant lesions	Mixed CHD cohorts; not direct obesity comparison

## Data Availability

No new data were created or analyzed in this study. Data sharing is not applicable to this article.
